# Similar Bacterial Communities among Different Populations of a Newly Emerging Invasive Species, *Tuta absoluta* (Meyrick)

**DOI:** 10.3390/insects13030252

**Published:** 2022-03-03

**Authors:** Hao Wang, Xiaoqing Xian, Yujuan Gu, Cristina Castañé, Judit Arnó, Suran Wu, Fanghao Wan, Wanxue Liu, Guifen Zhang, Yibo Zhang

**Affiliations:** 1 State Key Laboratory for Biology of Plant Diseases and Insect Pests, Institute of Plant Protection, Chinese Academy of Agricultural Sciences, Beijing 100193, China; wh691827@163.com (H.W.); xianxiaoqing@caas.cn (X.X.); wanfanghao@caas.cn (F.W.); liuwanxue@caas.cn (W.L.); zhangguifen@caas.cn (G.Z.); 2 Guangzhou Customs Technology Center, Laboratory for Plant Quarantine, Guangzhou 510623, China; guyj@iqtcnet.cn; 3 Sustainable Plant Protection Department, Institute for Research and Technology in Agriculture (IRTA), 08348 Cabrils, Barcelona, Spain; cristina.castane@irta.cat (C.C.); judit.arno@irta.cat (J.A.); 4 Institute of Tropical Bioscience and Biotechnology, Chinese Academy of Tropical Agricultural Sciences, Haikou 571101, China; wusuran@itbb.org.cn; 5 Agricultural Genome Institute at Shenzhen, Chinese Academy of Agricultural Sciences, Shenzhen 518120, China; 6 Scientific Observing and Experimental Station of Crop Pests in Guilin, Ministry of Agriculture, Guilin 541399, China

**Keywords:** *Tuta absoluta*, gut microbiota, geographical populations

## Abstract

**Simple Summary:**

As an invasive pest in China, the moth *Tuta absoluta* has spread extremely quickly, and now causes serious harm to the Chinese tomato industry. Understanding gut microbial diversity and composition can potentially identify the adaptive potential of introduced species. In this study, we found there were no significant differences in microbial diversity among three geographical populations, and the gut microbial compositions were similar among the Spanish, Xinjiang and Yunnan geographical populations.

**Abstract:**

Microorganisms in the guts of insects enhance the adaptability of their hosts with different lifestyles, or those that live in different habitats. *Tuta absoluta* is an invasive pest that is a serious threat to tomato production in China. It has quickly spread and colonized Xinjiang, Yunnan and other provinces and regions. We used Illumina HiSeq next generation sequencing of the 16S rRNA gene to study and analyze the composition and diversity of the gut microbiota of three geographical populations of *T. absoluta*. At the phylum level, the most common bacteria in *T. absoluta* across all three geographical populations were Proteobacteria and Firmicutes. An uncultured bacterium in the Enterobacteriaceae was the dominant bacterial genus in the *T. absoluta* gut microbiotas. There were no significant differences in alpha diversity metrics among the Spanish, Yunnan and Xinjiang populations. The structures of the gut microbiota of the three populations were similar based on PCoA and NMDS results. The results confirmed that the microbial structures of *T. absoluta* from different regions were similar.

## 1. Introduction

The abundant and diverse gut microbial communities of insects are often adapted to their host gut environment, with some even developing coevolutionary associations with their hosts via sophisticated symbiotic interactions [1,2,3]. Insect guts provide habitats for microbes which, in turn, can influence many aspects of the hosts’ biology. The main functions of the gut microbiota in insects include nutrition acquisition, food digestion, immunity, and defense detoxification, as well as promoting growth, development, and breeding [4,5,6].

Phytophagous lepidopterans, including butterflies and moths, are among the most widespread and diverse taxa of insects on Earth, including approximately 160,000 described species in 47 superfamilies [7]. Many of them are major agricultural pests that feed either externally on leaf tissue (macrolepidopterans), or internally on the mesophyll layer of the stem or leaf (microlepidopterans) [8]. Gut microbiota may aid in the digestion and uptake of nutrients from plants [9,10]. Hence, the gut microbiota of herbivorous lepidopterans has received increasing interest and study, especially through the use of next-generation sequencing technologies [3,11,12,13].

Lepidopteran insect guts generally contain abundant microorganisms [14,15]. Several studies have investigated the intestinal bacterial communities of lepidopterans, such as *Helicoverpa armigera* (Hübner) (Lepidoptera: Noctuidae) [16,17,18], *Spodoptera littoralis* (Boisd.) (Lep.: Noctuidae) [19], *Spodoptera litura* Fab. (Lep.: Noctuidae) [20], *Spodoptera exigua* (Hübner) (Lep.: Noctuidae) [21], *Spodoptera frugiperda* (J.E. Smith) (Lep.: Noctuidae) [22,23], *Plutella xylostella* (L.) (Lep.: Yponomeutidae) [24,25,26], *Plodia interpunctella* (Hübner) (Lep.: Pyralidae) [27], *Grapholita molesta* (Busck) (Lep.: Tortricidae), *Cydia pomonella* (Linnaeus) (Lep.: Tortricidae) [28], and *Antheraea assamensis* (Lep.: Saturniidae) [29]. Meanwhile, gut microbiota of these insects are significantly affected by developmental stage [19,21], food/host plant type [26,27,28,30,31,32], geographical location [23,29], environment [12,33], and pathogen invasion [18]. Even though the microbiota of several distinct feeding guilds, including foliage feeder and fruit borer, have been explored, little is known about that of lepidopteran leaf miners.

*Tuta absoluta* (Meyrick) (Lepidoptera: Gelechiidae) is an invasive species and destructive pest worldwide [34]. Its larvae not only mine into and feed within the leaves of host plants, but also penetrate host fruits and axillary buds of young stems [35]. *T. absoluta* originated in Peru, and was only reported from South American countries prior to 2006, when it was introduced to Spain through the importation of tomato fruits [34,36]. Then, this pest quickly spread in Europe, Africa, and Asia at 800 km per year with the trade of agricultural products [35]. By 2020, this pest had spread to 110 countries and regions [37]. Recently, the *T. absoluta* has invaded and established itself in China [38], spreading to Xinjiang, Yunnan, and other provinces [39]. Based on genetic population analyses, this pest could have been introduced from two different pathways: a southwest pathway from Myanmar into Yunnan Province, and a northwest pathway from Kazakhstan into Xinjiang Province [39]. *T. absoluta*, as a destructive invasive pest worldwide, has attracted considerable attention from many entomologists all over the world. However, no studies have focused on the environmental adaptability of the gut microbes in *T. absoluta* adults thus far. In addition, it is not clear whether the microbiota of adult insects influence their invasion potential or plant pest potential.

In this study, we examined the composition and diversity of bacterial communities from the guts of female *T. absoluta* from three different geographical populations using the Illumina HiSeq sequencing of bacterial 16S rRNA gene PCR amplicons. The goals of this work were to provide a reference for analyzing the sources of three geographical populations of *T. absoluta* (two from China and one from Spain) and estimate *Wolbachia* infections in *T. absoluta* populations. These results will aid the bettering our understanding of the adaptation of a lepidopteran leaf miner pest to different geographical environments.

## 2. Materials and Methods

### 2.1. Sample Collection

Adults of *T. absoluta* were collected from Yunnan and Xinjiang, China, and Barcelona, Spain, from December 2019 to July 2020 (Table 1). The transport of the Spanish population into China met the legislative and security requirements of the two countries. The intestinal contents of approximately 20 insects from each geographical population were treated as one replicate; five replicates were prepared for each population. A sufficient number of freshly emerged female adults of *T. absoluta* were selected from each geographic group. The insects were surface sterilized with 75% alcohol 3 times for 1 min each and washed with sterile water more than 5 times. The complete gut tract was excised, and the midgut and hindgut were separated, placed in 1.5 mL centrifuge tubes with sterile water, and then stored in the laboratory at −80 °C for later use.

### 2.2. DNA Extraction and Sequencing

DNA was extracted from approximately 20 intestinal contents (per replicate) of *T. absoluta* using a TGuide S96 Magnetic Soil/Stool DNA Kit (TIANGEN, Beijing, China) according to the manufacturer’s protocol. The V3 + V4 region of 16S rDNA was amplified with the specific primers 338F (5′-ACTCCTACGGGAGGCAGCA-3′) and 806R (5′-GGACTACHVGGGTWTCTAAT-3′) [40]. The PCR conditions were as follows: 95 °C for 5 min, followed by 25 cycles of 95 °C for 30 s, 50 °C for 30 s and 72 °C for 40 s, and a final extension at 72 °C for 7 min. PCR products were purified using VAHTSTM DNA clean beads (Vazyme, Nanjing, China) as a template by Solexa PCR with 98 °C for 30 s, followed by 10 cycles of 98 °C for 10 s, 65 °C for 30 s and 72 °C for 30 s, and a final extension at 72 °C for 5 min. Then, the Solexa PCR products were used to construct libraries by Biomarker Technologies Company (Beijing, China).

### 2.3. Bioinformatics Analysis

After sequencing, the original data were spliced by FLASH [41] (version 1.2.7). The raw reads obtained by sequencing were filtered by using Trimmomatic [42] (version 0.33), and then Cutadapt [43] (version 1.9.1) was employed to identify and remove primer sequences, thus obtaining clean reads without primer sequences. The clean reads were spliced using USEARCH [44] (version 10.0), and then the spliced data were filtered according to the length range of different regions. The identification and removal of chimeras were carried out by UCHIME [45] (version 8.1), and the final effective reads were generated. Then, the average length, GC content, Q20 (%) [quality value (>20)/the total bases], Q30 (%) [quality value (>30)/the total bases], and effective ratio [effective reads/raw reads] were calculated. The high-quality sequences with 97% or greater similarities were identified as operational taxonomic units (OTUs) by USEARCH [44] (version 10.0). Since *Wolbachia* is an intracellular, maternally transmitted endosymbiont, the OTU sequences were screened and analyzed to remove *Wolbachia* sequences.

### 2.4. Diversity Analyses

The ACE, Chao1, Shannon and Simpson indices were evaluated using QIIME2 [46] and were analyzed with one-way analysis of variance (ANOVA) at a 0.05 level of significance by using IBM SPSS Statistics 26. For beta diversity analysis, the binary Jaccard algorithm was used to calculate the distance between samples. Beta diversity was analyzed by principal coordinate analysis [47] (PCoA) and nonmetric multidimensional scaling [48] (NMDS). Analysis of similarities (ANOSIM) was performed with the vegan package in the R environment.

### 2.5. Phylogenetic Analysis of Wolbachia

To compare *Wolbachia* infections in *T. absoluta* species and other insect species, a phylogenetic tree was constructed using the maximum likelihood (ML) method in MEGA (version 7.0) (https://www.megasoftware.net/ accessed on 24 November 2021). Bootstrap analysis was performed with 1000 replicates. The 16S rRNA gene of *Wolbachia* from *T. absoluta* was obtained by Biomarker Technologies Company (Beijing, China). The other sequences were downloaded from GenBank.

## 3. Results

### 3.1. Sequencing Quality (OTU) and Venn Analyses

**Figure 1:**
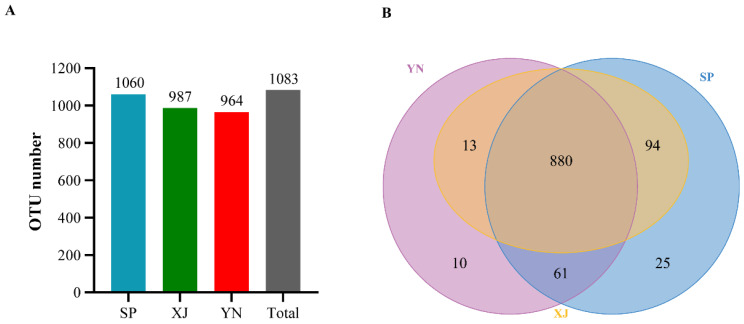
The results of OTU and Venn analyses of *T. absoluta* from different geographical populations. Abbreviations: SP, Spain; XJ, Xinjiang; YN, Yunnan. (**A**) Statistics on the OTU number in each sample. (**B**) Venn diagram of OTUs.

A total of 1,207,610 pairs of reads were obtained from 15 samples. After quality control and the splicing of double-terminal reads, a total of 1,193,147 clean reads were generated, and a minimum of 78,827 and an average of 79,543 clean reads were generated for each sample. The sequences were clustered into OTUs with a 97% identity, and a total of 1083 OTUs were obtained after removing *Wolbachia* (Figure 1A).

Figure 1 shows that 1060, 987 and 964 OTUs were obtained from three geographical populations of Spain (SP), Xinjiang (XJ) and Yunnan (YN), respectively (Figure 1A). Among these OTUs, 880 OTUs were shared among the gut microbiotas of the three geographical populations. There were 25 and 10 OTUs specific to the Spanish (SP) and Yunnan (YN) populations, respectively (Figure 1B).

### 3.2. Bacterial Alpha Diversity of the Gut Microbiota of T. absoluta

**Figure 2:**
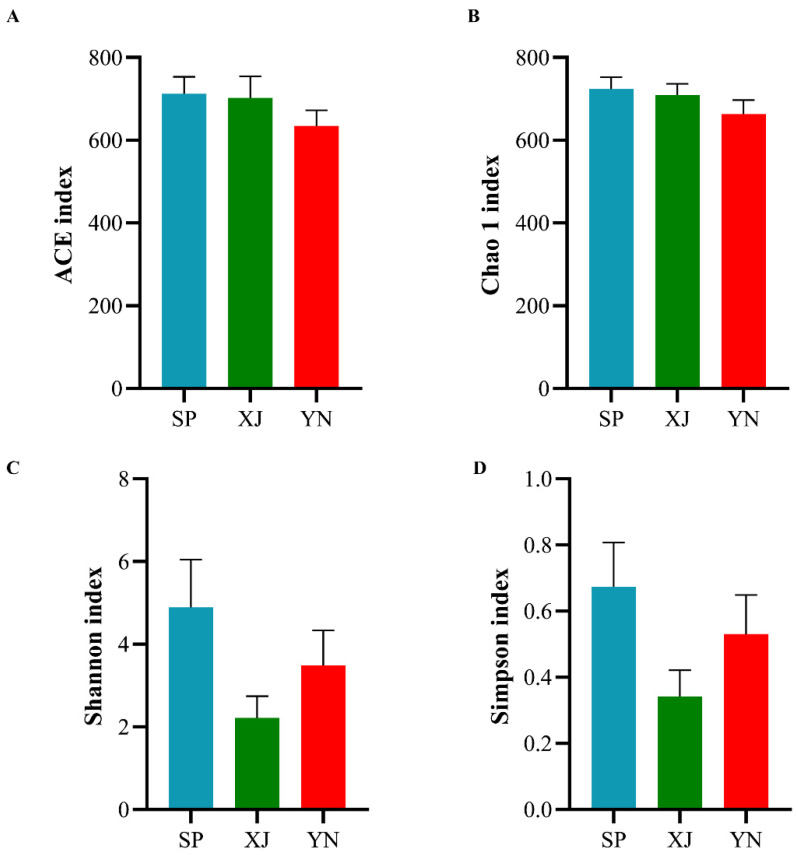
Analysis of alpha diversity indices of gut bacteria in *T. absoluta* from different geographical populations. Abbreviations: SP, Spain; XJ, Xinjiang; YN, Yunnan. (**A**) ACE index. (**B**) Chao 1 index. (**C**) Shannon index. (**D**) Simpson index.

The ACE index, Chao1 index, Shannon index and Simpson index were used to further analyze species diversity and richness. As shown in Figure 2, among the three groups, the Spanish population exhibited the highest Shannon and Simpson indices, while the lowest values were observed in the Xinjiang population. However, there were no significant differences in microbial diversity (*P*_Shannon_ = 0.142; *P*_Simpson_ = 0.156). The ACE and Chao1 microbial richness indices were also not significantly different between the geographical populations (*P*_ACE_ = 0.419; *P*_Chao1_ = 0.370). The species richness of the Spanish population species richness appeared to be higher than that of the Xinjiang population and Yunnan population.

### 3.3. Analysis of the Composition and Structure of the Gut Microbiota in T. absoluta from Different Geographical Populations

**Figure 3:**
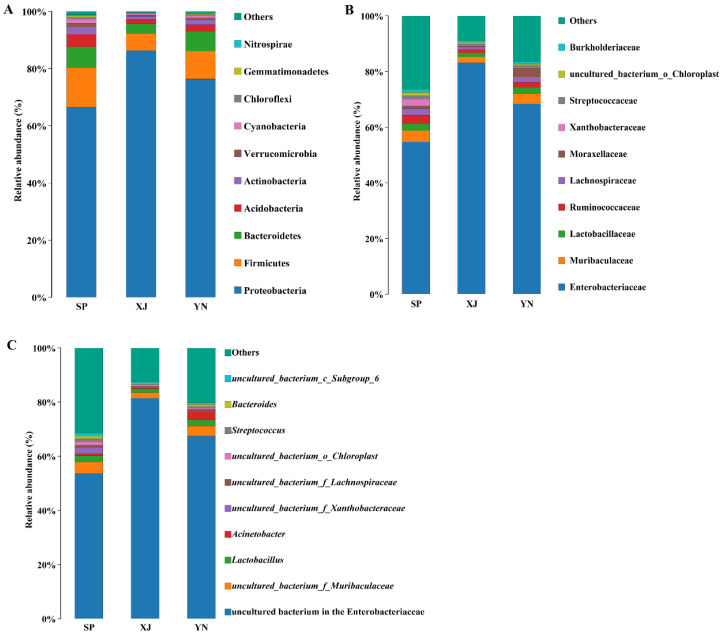
Species distribution in all samples. Abbreviations: SP, Spain; XJ, Xinjiang; YN, Yunnan. (**A**) Relative abundance of the top 10 bacteria at the phylum level in *T. absoluta* from three geographical populations. (**B**) Relative abundance of the top 10 bacteria at the family level. (**C**) Relative abundance of the top 10 bacteria at the genus level.

The microbial community composition of *T. absoluta* gut samples at the phylum, family, and genus level are shown in Figure 3. At the phylum level, the dominant gut bacteria of *T. absoluta* from the Spanish, Xinjiang, and Yunnan populations were members of Proteobacteria followed by Firmicutes, Bacteroidetes, Acidobacteria, Actinobacteria, Verrucomicrobia, Cyanobacteria, Chloroflexi, Gemmatimonadetes, and Nitrospirae (Figure 3A). At the family level, Enterobacteriaceae and Muribaculaceae were dominant in the Spanish, Xinjiang, and Yunnan populations (Figure 3B). When we kept sequences in *Wolbachia*, they accounted for 11.92% from Spain (SP), 4.77% from Xinjiang (XJ), and 10.59% from Yunnan (YN) in the genus level, respectively (Figure S1). However, *Wolbachia* is an endosymbiont, and we removed *Wolbachia* from the composition of the gut microbiota analysis. As shown in Figure 3C, an uncultured bacterium in the Enterobacteriaceae was the dominant genus of the gut microbial community in the three populations. The next most dominant was *uncultured*_*bacterium*_*f*_*Muribaculaceae*. The compositions and structures of the gut microbiota of *T. absoluta* from different geographical populations were similar.

### 3.4. The Bacterial Community Composition of the Gut Microbiota of T. absoluta

**Figure 4:**
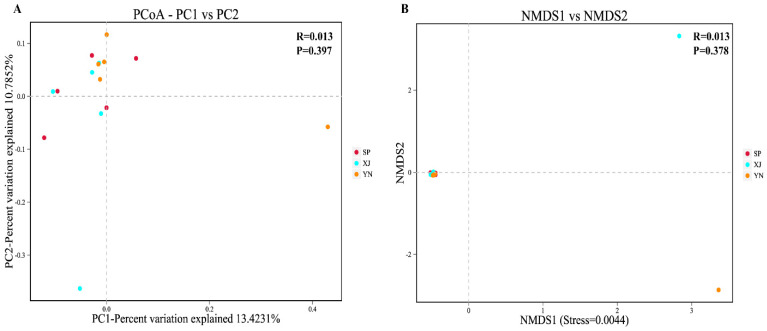
Beta diversity analysis of *T. absoluta* from different locations. Abbreviations: SP, Spain; XJ, Xinjiang; YN, Yunnan. (**A**) Principal coordinates analysis (PCoA) and (**B**) nonmetric multidimensional scaling (NMDS) based on the binary Jaccard distance. The reliability of NMDS with a stress value less than 0.2 is acceptable.

The dots separated by shorter distances shared higher similarity in the coordinate system (Figure 4A,B). PCoA and NMDS based on the binary Jaccard algorithm revealed that the compositions and structures of the gut microbiota in *T. absoluta* from Spanish, Yunnan, and Xinjiang populations overlapped, indicating that the microbial communities were very similar to each other. There was no significant difference in the gut microbiota among the three geographical populations of *T. absoluta* at the OTU level based on ANOSIM analysis (*P* = 0.397 for PCoA, *P* = 0.378 for NMDS).

### 3.5. Phylogenetic Analysis of Wolbachia

**Figure 5:**
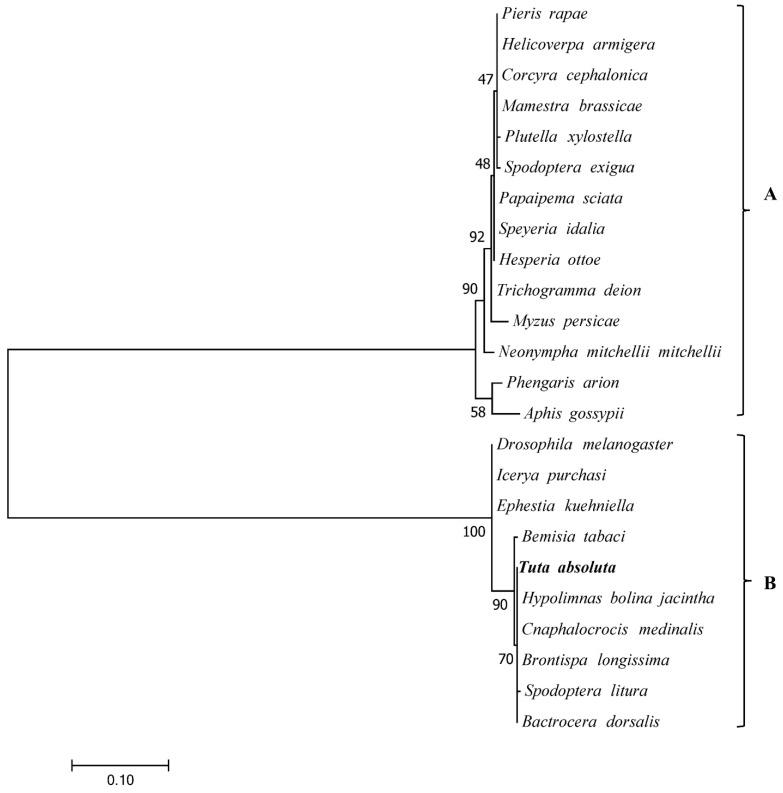
Phylogenetic tree of *Wolbachia* infecting in *T. absoluta* and other insect species. The GenBank accession numbers of the sequences of other species are as follows: *Pieris rapae* (EU753171); *Helicoverpa armigera* (EU753172); *Corcyra cephalonica* (EU753167); *Mamestra brassicae* (EU753175); *Plutella xylostella* (EU753169); *Spodoptera exigua* (EU753173); *Papaipema sciata* (KJ125432); *Speyeria idalia* (KJ125433); *Hesperia ottoe* (KJ125435); *Trichogramma deion* (L02888); *Myzus persicae* (MG707970); *Neonympha mitchellii mitchellii* (KJ125431); *Phengaris arion* (KM517520); *Aphis gossypii* (MG707918); *Drosophila melanogaster* (AB360385); *Ephestia kuehniella* (AB360384); *Bemisia tabaci* (OK042302); *Hypolimnas bolina jacintha* (AB085178.1); *Cnaphalocrocis medinalis* (HQ336509); *Brontispa longissimi* (L02888); *Spodoptera litura* (KC915389); and *Bactrocera dorsalis* (MK860779).

We constructed a phylogenetic tree of 24 *Wolbachia* sequences by using the ML method to analyze the evolutionary relationships of *Wolbachia* from *T. absoluta* and other arthropod hosts (Figure 5), and all sequences were divided into two supergroups (A and B). *T. absoluta* in this research were a member of *Wolbachia* supergroup B.

## 4. Discussion

*T. absoluta* is a destructive pest of tomato worldwide. In the present study, the composition and diversity of the gut bacteria of *T. absoluta* from three different geographical regions were studied to promote further research on whether these bacteria influence invasion potential or pest status. The results revealed abundant and diverse bacteria in the gut of *T. absoluta*.

Alpha diversity reflects the species richness and diversity of a single sample [49]. Based on the alpha diversity metrics, there were no significant differences among the three geographical populations. Our result was consistent with the findings of some previous research. Ugwu et al. [23] found no significant differences in the microbial diversity and species richness of different geographical populations of *Spodoptera frugiperda*. In contrast, Gandotra et al. [29] reported that *Antheraea assamensis* from the Titabar region had a significantly higher Shannon–Wiener diversity index than that in the other regions. Bacterial diversity (Shannon index) varied significantly between *Holotrichia parallela* larvae from different locations [50].

In addition, the microbial community structure relationships among the Spanish (SP), Yunnan (YN), and Xinjiang (XJ) populations were similar, which could have two explanations. First, tomato is the preferred host plant in the different geographical populations of *T. absoluta*. The *T. absoluta* diet is narrow, with a preference for tomato (fresh market and cherry tomatoes) over other solanaceous plants in China [39]. In addition, studies of microbial communities of *Aphis gossyphii* from different plants and regions in China uncovered no significant correlation between the geographical distance between sampling sites and the Bray–Curtis differences of symbiotic or secondary symbiotic communities, and determined that host plants may have influenced the composition of the associated symbionts [51]. Moreover, Ugwu et al. [29] revealed that *Spodoptera frugiperda* from different geographical regions did not differ in the larval gut microbiome. However, Gong et al. [52] suggested that environmental variability can influence the gut microbiota of the two fruit moth pests. The object of our study was the gut microbiota of adults from different geographical populations. Because the lepidopteran larvae consume large kinds of host plants during development their guts may contain many undigested host plants. Further studies will examine more species of host plants and different environments to analyze the adaptability of the larvae of *T. absolute* gut microbiota. The second reason may be that the three geographical populations have the same origin. As a newly emerging invasive pest, *T. absoluta* was first detected only a few months apart in the Xinjiang and Yunnan provinces [38]. Zhang et al. [39] speculated that neither of these provinces was the source of the introduction to the other province. Based on the microbiome results, we propose that Yunnan and Xinjiang populations could have originated from the Spanish population. Further research will be combined with genetic studies to test this hypothesis about the origin of *T. absoluta*.

The gut microbiota of *T. absoluta* was dominated by Proteobacteria and Firmicutes at the phylum level, similar to the results of previous studies on lepidopteran insects, including *Spodoptera littoralis* [19], *Helicoverpa armigera* [16], *Plutella xylostella* (L.) [24,53], *Brithys crini* [54], and *Spodoptera frugiperda* [55]. A comparison of the gut bacteria of 30 species of lepidopterans revealed that the most dominant phylum was Proteobacteria [56]. In this study, family Enterobacteriaceae, a member of the Proteobacteria, was dominant in all geographical populations. Enterobacteriaceae are widely distributed in insects and have an important function. An increased abundance of Enterobacteriaceae can significantly reduce the mortality of host insects, thus extending host insect longevity [57]. Enterobacteriaceae can also aid in insects’ nitrogen and carbon metabolism [58,59,60], resistance to pathogenic bacteria and parasites [61], and courtship and reproduction [28]. Interestingly, the microbial richness of the Xinjiang (XJ) population was lower than that of the Spanish (SP) population and the Yunnan (YN) population at the family level, except for Enterobacteriaceae. We speculate that metabolic versatility can help insects adapt to unfavorable environments. This hypothesis can be tested with additional in-depth research in this area.

*Wolbachia* is found in many arthropods, especially insects [62]. Approximately 80% of lepidopteran insects are infected by *Wolbachia* [63]. As a facultative endosymbiont, *Wolbachia* has become one of the most widely studied endosymbionts due to its wide distribution and multiple regulatory effects on the host. Furthermore, *Wolbachia* induces the reproductive manipulation of hosts, including cytoplasmic incompatibility (CI), male killing, feminization, and parthenogenesis [64]. Most *Wolbachia* infecting lepidopteran insects belong to supergroup A or B, and most of them belong to supergroup B [65,66]. In the current study, the *Wolbachia* detected in the three different geographical populations of *T. absoluta* appeared to belong to supergroup B, which was consistent with previous results in a Brazilian population and Iranian and Turkish populations [67,68].

*T. absoluta* is an invasive pest worldwide and has been found to have parthenogenesis [69,70]. This may be related to the symbiosis of *T. absoluta* with a specific strain of *Wolbachia* [67]. However, the parthenogenesis of the population of *T. absoluta* in Brazil is independent of infection by *Wolbachia* [67]. To date, parthenogenesis has not been found for *T. absoluta* in China, a newly invaded country. We do not know whether the presence of *Wolbachia* in *T. absoluta* is responsible for this reproductive behavior. Further studies with the application of molecular and genetic techniques and enhanced international collaboration will help answer this question.

## 5. Conclusions

In summary, our results showed the diversity and composition of the *T. absoluta* gut microbiota from different regions. We confirmed that Proteobacteria and Firmicutes were the dominant bacteria at the phylum level. We also found that the gut microbial structures of *T. absoluta* populations from different regions were similar. Our results may shed light on the invasiveness and adaptability of *T. absoluta* from the perspective of gut microbiota.

## Figures and Tables

**Table 1:** Sampling locations and dates for the populations of *Tuta absoluta*.

Population	No. Guts	Location (Collection Date)	Longitude and Latitude	Crop
Xinjiang	17	Yili, Xinjiang, China; XJ (July 2020)	81.7978, 43.1985	Tomato, field
	18			
	18			
	20			
	20			
Yunnan	17	Yuxi, Yunan, China; YN (June 2020)	102.5388, 24.3602	Tomato, field
	18			
	20			
	19			
	20			
Spain	18	Barcelona, Spain; SP (December 2019)	2.3806, 41.5655	Tomato, field
	18			
	19			
	21			
	20			

## Supplementary Materials

The following supporting information can be downloaded at: https://www.mdpi.com/article/10.3390/insects13030252/s1, Figure S1: Species distribution in all samples when kept *Wolbachia* in analysis.
