# Supplementary material for: Similar Bacterial Communities among Different Populations of a Newly Emerging Invasive Species, Tuta absoluta (Meyrick)
Source: Insects. 2022 Mar 3;13(3):252. doi: 10.3390/insects13030252 (PMC8951508; doi:10.3390/insects13030252)
Supplement: Supplementary file 1 [file insects-13-00252-s001.zip › insects-1543642-supplementary.pdf]

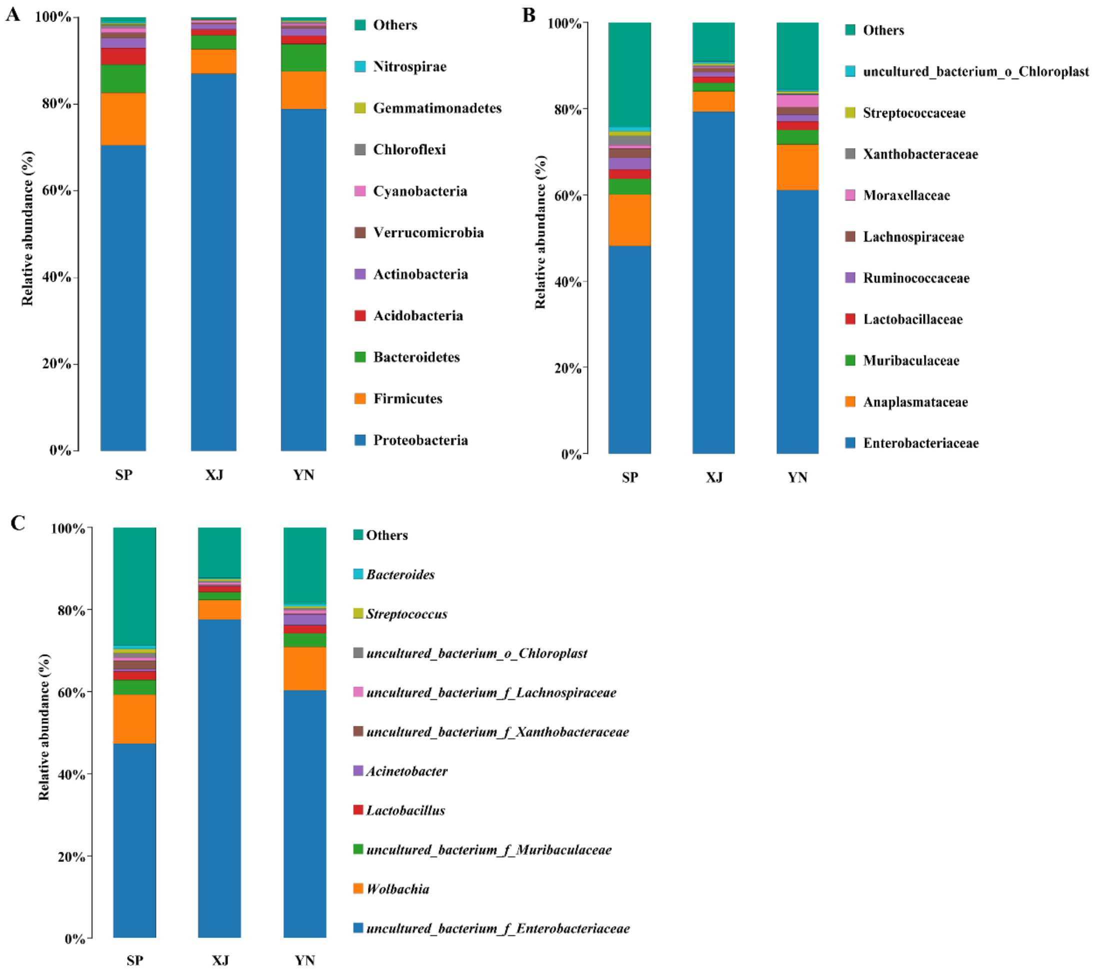

Figure S1. Species distribution in all samples when kept *Wolbachia* in analysis. Abbreviations: SP, Spain; XJ, Xinjiang; YN, Yunnan. (A) Relative abundance of the top 10 bacteria at the phylum level in *T. absoluta* from three geographical populations. (B) Relative abundance of the top 10 bacteria at the family level. (C) Relative abundance of the top 10 bacteria at the genus level.
